# Pulmonary Nodule Classification with Deep Convolutional Neural Networks on Computed Tomography Images

**DOI:** 10.1155/2016/6215085

**Published:** 2016-12-14

**Authors:** Wei Li, Peng Cao, Dazhe Zhao, Junbo Wang

**Affiliations:** ^1^Medical Image Computing Laboratory of Ministry of Education, Northeastern University, Shenyang 110819, China; ^2^College of Computer Science and Engineering, Northeastern University, Shenyang 110819, China; ^3^Neusoft Research Institute, Neusoft Corporation, Shenyang 110179, China

## Abstract

Computer aided detection (CAD) systems can assist radiologists by offering a second opinion on early diagnosis of lung cancer. Classification and feature representation play critical roles in false-positive reduction (FPR) in lung nodule CAD. We design a deep convolutional neural networks method for nodule classification, which has an advantage of autolearning representation and strong generalization ability. A specified network structure for nodule images is proposed to solve the recognition of three types of nodules, that is, solid, semisolid, and ground glass opacity (GGO). Deep convolutional neural networks are trained by 62,492 regions-of-interest (ROIs) samples including 40,772 nodules and 21,720 nonnodules from the Lung Image Database Consortium (LIDC) database. Experimental results demonstrate the effectiveness of the proposed method in terms of sensitivity and overall accuracy and that it consistently outperforms the competing methods.

## 1. Introduction

Lung cancer is becoming one of the main threats to human health at present in the world. The number of deaths caused due to lung cancer is more than prostate, colon, and breast cancers [1]. Early detection of solitary pulmonary nodules (SPNs) is an important clinical indication for early-stage lung cancer diagnosis because SPNs have high probabilities to become malignant nodules [2, 3]. SPNs refer to lung tissue abnormalities that are roughly spherical with round opacity and a diameter of up to 30 mm.

**Figure 1:**
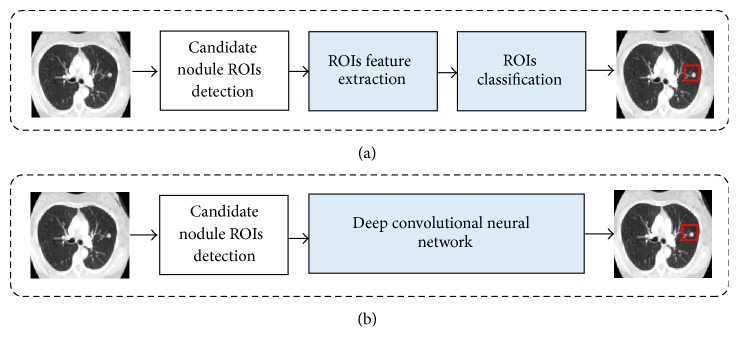
The main components in a general CAD system (a) and the main components in our work (b).

It is therefore an important task to develop computer aided detection (CAD) systems that can aid/enhance radiologist workflow and potentially reduce false-negative findings. CAD is a scheme that automatically detects suspicious lesions (i.e., nodule, polyps, and masses) in medical images of certain body parts and provides their locations to radiologists [4–6]. CAD has become one of the major research topics in medical imaging and diagnostic radiology and has been applied to various medical imaging modalities including computed tomography (CT) [7], magnetic resonance imaging (MRI) [8], and ultrasound imaging [9]. Generally, typical CAD systems for cancer detection and diagnosis (i.e., breast, lung, and polyp) cover four stages as depicted in Figure 1(a), including candidate nodule ROI (Region of Interest) detection, feature extraction, and nodule classification. The stages of feature extraction and nodule classification belong to the false-positive reduction step. Current CAD schemes for nodule characterization have achieved high sensitivity levels and would be able to improve radiologists' performance in the characterization of nodules in thin-section CT, whereas current schemes for nodule detection appear to report many false positives. It is because detection algorithms have high sensitivity that some nonnodule structures (e.g., blood vessels) are labeled as nodules inevitably in the initial nodule identification step. Since the radiologists must examine each identified object, it is highly desirable to eliminate these false positives (FPs) as much as possible while retaining the true positives (TPs). Therefore, significant effort is needed in order to improve the performance levels of current CAD schemes for nodule detection in thin-section CT.

The purpose of false-positive reduction is to remove these false positives (FPs) as much as possible while retaining a relatively high sensitivity [10, 11]. It is a binary classification between the nodule and nonnodule, aiming to develop new methods in order to accurately distinguish suspicious regions, leading to significant reduction of FPs with machine learning techniques. The false-positive reduction step, or classification step, the aim of which is to learn a system capable of the prediction of the unknown output class of a previously unseen suspicious nodule with a good generalization ability, is a critical part in the lung nodule detection system. Classification plays an important role in the reduction of false positives in lung computer aided detection and diagnosis methods. Deep learning can be used for both classification and feature learning in various fields such as computer vision and speech. In our work, a deep convolutional neural network is proposed for pulmonary nodule classification using the LIDC database. The method used in CAD system replaces the two components of feature extraction and classification. The input of deep convolutional neural networks in this work is ROI pixel data directly without feature extraction and selection. Compared with the traditional methods, the approach in our work has four advantages as follows.

- The representation of nodule ROI is critical for discrimination between true nodule and false nodule. However, it is difficult to obtain good feature representations by human efforts. Our method can learn a good feature representation of ROI without feature extraction and selection.
- Our method takes advantage of the relationships between the internal region and external region of ROI, so as to learn more discriminative knowledge for false-positive reduction.
- Our method can be executed based on the center of the ROI rather than the whole ROI region. Therefore, there is no necessity to obtain the exact margin of the nodules detected in the first step of CAD system.
- The neural networks are trained by large scale ROIs data with nodules and nonnodules more than 60 thousand which are the largest in our knowledge. So the neural network is capable of recognizing a wide range of representations of nodules.

The rest of the paper is organized as follows.  Section 2 analyzes the related works. The methodology to recognize nodules is described in Section 3. The experimental results obtained are discussed in Section 4. We conclude this paper in Section 5.

## 2. Related Work

At present, a lot of works have been done in pulmonary nodule recognition research. The pulmonary nodule recognition involves nodule candidate detection [12] and false-positive reduction [13]. The traditional approaches of false-positive reduction have successive steps: feature extraction [14, 15] and classifier model construction [10, 16]. The most effective features which can be used for classification for lung CT images are, for example, shape, intensity, texture, geometric, gradient, and wavelet. Texture features as Haralick, Gabor, and Local Binary Patterns are used to analyze lung nodules in [17]. MR8, LBP (Local Binary Patterns), Sift descriptor, and MHOG (Multiorientation Histogram of Oriented Gradients) are used for the feature extraction process in [18], and the SURF (Speed-Up Robust Feature) and the LBP descriptors are used to generate the features that describe the texture of common lung nodules in [19]. Mohammad applied an improved LBP feature in lung nodule detection which is robust for noise [20]. Sui et al. used 2D features of circularity, elongation, compactness, moment, and 3D features as surface-area, volume, sphericity, and centroid-offset for lung nodule recognition [21]. Although the feature is well and comprehensively designed, the classifiers in the third step of CAD system still show their deficiencies on classifying the nodule images precisely. Generally speaking, the classifiers are supervised learning approaches in machine learning domain, such as SVM, *k*-nearest neighbor (*k*-NN), artificial neural networks (ANNs), and decision tree which have been used in lung nodule classification [22]. In addition, Zhang et al. designed a classifier in a semisupervised way exploring the information from unlabeled images [23]. In order to improve the ensemble classification advantage in lung nodule recognition task, a random forest algorithm with a structure for a hybrid random forest aided by clustering is described in [24]. The imbalance distribution between the amounts of nodule and nonnodule candidates comes out in mostly datasets. Sui et al. present a novel SVM classifier combined with random undersampling and SMOTE for lung nodule recognition [21]. Cao et al. extend the random subspace method to a novel Cost Sensitive Adaptive Random Subspace (CSARS) ensemble to overcome imbalanced data classification [10].

In recent years, deep artificial neural networks have won numerous contests in pattern recognition and machine learning. Convolutional neural networks (CNNs) constitute one such class of models [30]. In 2012, an ensemble CNNs approach achieved the best results on the ImageNet classification benchmark, which is popular in the computer vision community [31]. There has also been popular latest research in area of medical imaging using deep learning with promising results. Suk et al. propose a novel latent and shared feature representation of neuroimaging data of brain using Deep Boltzmann Machine (DBM) for AD/MDC diagnosis [32]. Wu et al. use deep feature learning for deformable registration of brain MR images to improve image registration by using deep features [33]. Xu et al. present the effectiveness of using deep neural networks (DNNs) for feature extraction in medical image analysis as a supervised approach [34]. Kumar et al. propose a CAD system which uses deep features extracted from an autoencoder to classify lung nodules as either malignant or benign on LIDC database, which is similar to our work [35]. Convolutional neural networks have performed better than DBNs by themselves in current literature on benchmark computer vision datasets. The CNNs have attracted considerable interest in machine learning since they have strong representation ability in learning useful features from input data in recent years [36]. Moreover, to the best of our knowledge there has been no work that uses deep convolutional neural networks for lung nodule classification. Therefore, we evaluate the CNN on the computer aided lung nodule.

## 3. Proposed Method

### 3.1. Data

The dataset used in this work is the LIDC-IDRI dataset [37], consisting of 1010 thoracic CT scans with nodule size reports and diagnosis reports that serve as a medical imaging research resource. Four radiologists reviewed each scan using two blinded phases. The results of each radiologist's unblinded review were compiled to form the final unblinded review. The LIDC radiologists' annotations include freehand outlines of nodules ≥ 3 mm in diameter on each CT slice in which the nodules are visible, along with the subjective ratings on a five- or six-point scale of the following pathologic features: calcification, internal structure, subtlety, lobulation, margins, sphericity, malignancy, texture, and spiculation. The annotations also include a single mark (an approximate centroid) of nodules ≤ 3 mm in diameter as well as nonnodules ≥ 3 mm.

We included nodules with their annotated centers from the nodule report. The average width and height of the nodule images are 14 pixels, and the median is 12 pixels. The nodules whose sizes are less than 32*∗*32 account for 95.33% of the overall data, and the percentage is 99.991% for less than 64*∗*64 size of nodules.

In the first step of the ROI extraction, the geometric center is computed by the region margin marked in the database. Then region size is determined whether it is larger than 32*∗*32. The 32*∗*32 rectangle region is segmented with the same geometric of the marked region if its size is less than 32*∗*32. Otherwise, a larger size of 64*∗*64 is obtained as a candidate ROI and then is downsampled to 32*∗*32 size finally. There are nonnodule annotated regions extracted by the same way to form the negative sample during the training and testing process. In order to evaluate the effectiveness of the neural networks for different image sizes, dataset is also made with 64*∗*64 size using the same procedure. As a result, a total of 62,492 ROI image patches are extracted from 1,013 LIDC lung image cases containing 40,772 nodules and 21,720 nonnodules.

### 3.2. Convolutional Neural Network Construction

**Figure 2:**
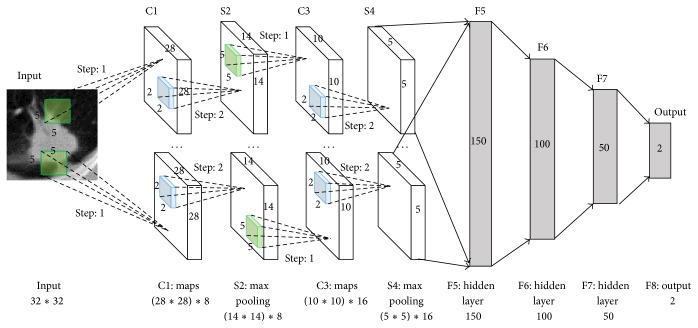
Architecture of our CNN for nodule recognition. The input data is ROI image pixels as a 1024-dimensional vector, and the number of output neurons of the network is 2 (nodule: 1 and nonnodule: 0). The numbers of neurons in the other layers are set to 6272, 1568, 1600, 250, 150, 100, and 50.

In computer vision, deep convolutional neural networks (CNNs) have been introduced because they can simulate the behavior of the human vision system and learn hierarchical features, allowing object local invariance and robustness to translation and distortion in the model [36]. CNNs are an alternative type of neural network that can be used to model spatial and temporal correlation while reducing translational variance in signals. The deep convolutional neural networks are built based on the size of input images. The structures of networks are different according to the different image size. A deep CNN proposed in this paper is constructed on 32*∗*32 image ROI data as an example presented in Figure 2.

The convolutional neural networks have two convolutional layers and there is a downsampling layer behind the convolutional layer. Fully connected layers are appended to the last downsampling layer. The first convolutional layer contains 8 feature maps, and the second has 16 ones. The kernel size is 5*∗*5 in all convolutional layers and the step of kernel is 1. The kernel size is 2*∗*2 for all the downsampling layers and the step is 2. The first fully connected layer contains 150 nodes and there are 100 nodes in the second fully connected layer. There are 50 nodes in the third fully connected layer and the last layer only has two nodes which are presented as output probabilities of nodule and nonnodule. The ROI region can be recognized as nodule or nonnodule by the output probabilities. In the same way, the convolutional neural networks can be constructed for 64*∗*64 size input image only and the convolution kernel size, convolution kernels moving step, feature map, and the number of nodes are adjusted which are not discussed here.

### 3.3. Neural Network Training

The deep CNNs described in above section are trained by the LIDC ROI image set extracted in Section 3.1. Firstly, the random initialization of the network weights is conducted and then ROI images are normalized as input into the neural network. At the training stage, the images entered into the network are with labels; that is, each ROI area is known as pulmonary nodules or not. Given each layer in the network input as *X* and output as *Y*, the current layer as the convolutional or fully connected layer is calculated as *Y* = max⁡(0, *ωX* + *B*), where *ω* is the current layer weights corresponding to each node and *B* is the bias parameter. The formulation is *Y* = max⁡(*X*) for the downsampling layers. The output layer is a softmax layer that predicts the probability of the nodule class. Two probabilities are obtained in the output layer after computing operations followed as above descriptions from input image data. The new weights values can be updated by backpropagation algorithm using the two probabilities and the label data with 0 or 1 [16]. The training process is terminated when the accuracy is up to predetermined value or the convergence condition. Finally, the evaluation is conducted on the testing data with the trained model.

## 4. Experiments

**Figure 3:**
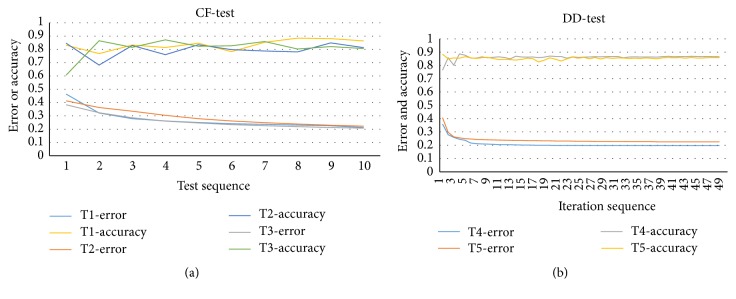
The classification performance with respect to error and accuracy with iteration number.

The experimental evaluations are conducted on LIDC database. The test scheme is designed as two different strategies. One is 10-fold cross-validation (CF-test) and the other is that the dataset is divided into the training data (85.7%) and testing data (DD-test). Since all the previous works are based on the manually designed features while the proposed approach in this paper is based on feature learning and nodule recognition by deep convolutional neural networks, it is not possible to directly compare our method with them on the same LIDC dataset. All experiments are conducted on a desktop computer with Intel Core 2 CPU of 2.80 GHz, 8 GB memory, and Windows 7. The algorithm is implemented by C++ in Microsoft Visual Studio 2010. The performance is shown as in Table 1 with both CF-test and DD-test. The tests T1, T2, and T3 are used by the strategy of CF-test and the parameters of convolutional map size and momentum for weight updating are set as 6 and 0.9. The learning rates in T1 and T2 are 0.0005 while T3 is 0.001. The image sizes in T2 and T3 are 64*∗*64 and the other ones are 32*∗*32. The tests T4 and T5 are used by the strategy of DD-test where the momentum and learning rate are set as 0.95 and 0.0005, respectively. However, the convolutional map size is set to 6 for T4 and 8 for T5 test. In CF-test, the learning rate keeps unchanged in the entire training process. However, the learning rates in T4 and T5 tests are decreased by 5/6 of last iteration once the value of precise up to 0.85.  Figure 3(a) shows that the performance of accuracy and error trend in CF-test and the same evaluation result is presented in Figure 3(b) which has the maximum iteration to 50.

**Figure 4:**
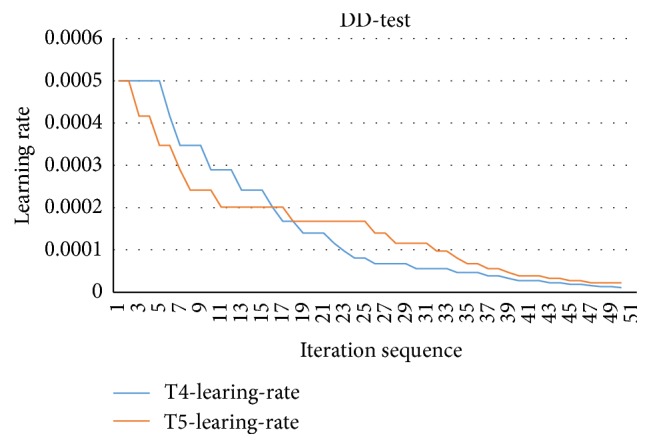
The learning rate changes in training process.

The learning rate is changing in the DD-test benchmark which is shown in Figure 4. In DD-test evaluation, the training process is conducted on the training dataset which will be shuffled at the beginning of training at every iteration, and then the model is applied on the testing dataset which is not changed in the entire testing time. Therefore, a new evaluation result is obtained in each iteration. From Table 1, the deep convolutional neural networks obtain a promising performance on pulmonary nodule recognition on CT images. The best accuracy is 0.864 and sensitivity is 0.890. The results also demonstrate that the larger value of the momentum and learning rate can achieve a fast convergence performance.

The results shown in Figures 3 and 4 demonstrate that the learning rate converges more smoothly compared with CF-test. Although the change of accuracy is large at the top iteration in CF-test, the error is increasing in training gradually and the whole networks are stable in the last. However, the performance with respect to error and accuracy becomes much more stable after several iterations. This behavior is correlated with the change of learning rate, because when the network obtains an optimal point then the training process gets stable. Overall, the deep convolutional neural network shows its stability and robustness in the training process. Moreover, the CNN framework is effective and efficient in classification.

In order to show the performance of the deep learning based method, we compared it with the state-of-the-art methods designed for lung nodule detection. The result is shown in Table 2. Strictly speaking, it is hard to compare to other reported works on the lung nodule detection problem. This is because most work does not employ the whole LIDC datasets. From the results in Table 2, our empirical results are very encouraging and have demonstrated the promise of the proposed method in the lung nodule detection with respect to sensitivity and FP/exam.

## 5. Conclusions

In this paper, a method of pulmonary nodule recognition using deep convolutional neural networks is presented. The deep convolutional neural network can take advantage of the training dataset to enable the algorithm to automatically select the best representation as the feature representation of the image. Through the training of the training dataset, the approach obtains much more general characteristics of pulmonary nodules and higher accuracy while retaining relatively better robustness. We plan to extend the proposed method to be capable of benign and malignant classification in the future. The algorithm will be accelerated by GPU computing for convolution operation.

## Figures and Tables

**Table 1:** Performance for CF-test and DD-test.

TID	Accuracy	Sensitivity	FP/exam	*F*-measure	Time (s)
T1	0.855	0.855	4.276	0.870	5,236
T2	0.849	0.866	**3.957**	0.858	28,761
T3	0.857	0.871	4.459	0.864	19,302
T4	**0.864**	**0.890**	5.546	**0.877**	19,993
T5	0.843	0.871	5.540	0.857	21,920

**Table 2:** Comparison of studies on nodule detection.

Work	Database	Cases	Sensitivity (%)	FP/exam
Proposed method	LIDC	1010	87.1	4.622
Netto et al. [25]	LIDC	29	85.9	0.138
Pei et al. [26]	LIDC	30	100	8.4
Pu et al. [27]	LIDC	52	81.5	6.5
Namin et al. [28]	LIDC	63	88.0	10.3
Messay et al. [29]	LIDC	84	82.66	3

## References

[1] Bach P. B., Mirkin J. N., Oliver T. K. (2012). Benefits and harms of CT screening for lung cancer: a systematic review. *JAMA*.

[2] Winer-Muram H. T. (2006). The solitary pulmonary nodule. *Radiology*.

[3] Suzuki K., Li F., Sone S., Doi K. (2005). Computer-aided diagnostic scheme for distinction between benign and malignant nodules in thoracic low-dose CT by use of massive training artificial neural network. *IEEE Transactions on Medical Imaging*.

[4] Suzuki K. (2012). A review of computer-aided diagnosis in thoracic and colonic imaging. *Quantitative Imaging in Medicine and Surgery*.

[5] Tan M., Deklerck R., Jansen B., Bister M., Cornelis J. (2011). A novel computer-aided lung nodule detection system for CT images. *Medical Physics*.

[6] Gurcan M. N., Sahiner B., Petrick N. (2002). Lung nodule detection on thoracic computed tomography images: preliminary evaluation of a computer-aided diagnosis system. *Medical Physics*.

[7] Peng S.-H., Kim D.-H., Lee S.-L., Lim M.-K. (2010). Texture feature extraction based on a uniformity estimation method for local brightness and structure in chest CT images. *Computers in Biology and Medicine*.

[8] Zhang Y., Wang S., Phillips P., Dong Z., Ji G., Yang J. (2015). Detection of Alzheimer's disease and mild cognitive impairment based on structural volumetric MR images using 3D-DWT and WTA-KSVM trained by PSOTVAC. *Biomedical Signal Processing and Control*.

[9] Chang C.-Y., Chen S.-J., Tsai M.-F. (2010). Application of support-vector-machine-based method for feature selection and classification of thyroid nodules in ultrasound images. *Pattern Recognition*.

[10] Cao P., Zhao D., Zaiane O. Cost sensitive adaptive random subspace ensemble for computer-aided nodule detection.

[11] Suzuki K., Shiraishi J., Abe H., MacMahon H., Doi K. (2005). False-positive reduction in computer-aided diagnostic scheme for detecting nodules in chest radiographs by means of massive training artificial neural network. *Academic Radiology*.

[12] Suzuki K. (2009). A supervised 'lesion-enhancement' filter by use of a massive-training artificial neural network (MTANN) in computer-aided diagnosis (CAD). *Physics in Medicine and Biology*.

[13] Suzuki K., Armato S. G., Li F., Sone S., Doi K. (2003). Massive training artificial neural network (MTANN) for reduction of false positives in computerized detection of lung nodules in low-dose computed tomography. *Medical Physics*.

[14] Ye X., Lin X., Dehmeshki J., Slabaugh G., Beddoe G. (2009). Shape-based computer-aided detection of lung nodules in thoracic CT images. *IEEE Transactions on Biomedical Engineering*.

[15] Paik D. S., Beaulieu C. F., Rubin G. D. (2004). Surface normal overlap: a computer-aided detection algorithm with application to colonic polyps and lung nodules in helical CT. *IEEE Transactions on Medical Imaging*.

[16] Sun T., Wang J., Li X. (2013). Comparative evaluation of support vector machines for computer aided diagnosis of lung cancer in CT based on a multi-dimensional data set. *Computer Methods and Programs in Biomedicine*.

[17] Han F., Wang H., Zhang G. (2014). Texture feature analysis for computer-aided diagnosis on pulmonary nodules. *Journal of Digital Imaging*.

[18] Zhang F., Song Y., Cai W. (2014). Lung nodule classification with multilevel patch-based context analysis. *IEEE Transactions on Biomedical Engineering*.

[19] Amal F., Asem A., James G. (2010). Feature-based lung nodule classification. *Advances in Visual Computing: 6th International Symposium, ISVC 2010, Las Vegas, NV, USA, November 29– December 1, 2010, Proceedings, Part III*.

[20] Mohammad H. S. (2014). Lung nodule detection based on noise robust local binary pattern. *International Journal of Scientific and Engineering Research*.

[21] Sui Y., Wei Y., Zhao D. (2015). Computer-aided lung nodule recognition by SVM classifier based on combination of random undersampling and SMOTE. *Computational and Mathematical Methods in Medicine*.

[22] Song Y., Cai W., Zhou Y., Feng D. D. (2013). Feature-based image patch approximation for lung tissue classification. *IEEE Transactions on Medical Imaging*.

[23] Zhang F., Song Y., Cai W. A ranking-based lung nodule image classification method using unlabeled image knowledge.

[24] Lee S. L. A., Kouzani A. Z., Hu E. J. (2010). Random forest based lung nodule classification aided by clustering. *Computerized Medical Imaging and Graphics*.

[25] Netto S. M. B., Silva A. C., Nunes R. A., Gattass M. (2012). Automatic segmentation of lung nodules with growing neural gas and support vector machine. *Computers in Biology and Medicine*.

[26] Pei X., Guo H., Dai J. Computerized detection of lung nodules in CT images by use of multiscale filters and geometrical constraint region growing.

[27] Pu J., Zheng B., Leader J. K., Wang X.-H., Gur D. (2008). An automated CT based lung nodule detection scheme using geometric analysis of signed distance field. *Medical Physics*.

[28] Namin S. T., Moghaddam H. A., Jafari R., Esmaeil-Zadeh M., Gity M. Automated detection and classification of pulmonary nodules in 3D thoracic CT images.

[29] Messay T., Hardie R. C., Rogers S. K. (2010). A new computationally efficient CAD system for pulmonary nodule detection in CT imagery. *Medical Image Analysis*.

[30] Alpher A., Fotheringham-Smy J. P. N. Convolutional networks and applications in vision.

[31] Krizhevsky A., Sutskever I., Hinton G. E. Imagenet classification with deep convolutional neural networks.

[32] Suk H.-I., Lee S.-W., Shen D. (2014). Hierarchical feature representation and multimodal fusion with deep learning for AD/MCI diagnosis. *NeuroImage*.

[33] Wu G., Kim M., Wang Q., Gao Y., Liao S., Shen D. (2013). Unsupervised deep feature learning for deformable registration of MR brain images. *Medical Image Computing and Computer-Assisted Intervention*.

[34] Xu Y., Mo T., Feng Q., Zhong P., Lai M., Chang E. I.-C. Deep learning of feature representation with multiple instance learning for medical image analysis.

[35] Kumar D., Wong A., Clausi D. A. Lung nodule classification using deep features in CT images.

[36] Yann L., Yoshua B. (1995). Convolutional networks for images, speech, and time series. *The Handbook of Brain Theory and Neural Networks*.

[37] Armato S. G., McLennan G., Bidaut L. (2011). The lung image database consortium (LIDC) and image database resource initiative (IDRI): a completed reference database of lung nodules on CT scans. *Medical Physics*.

